# A Versatile Method for Viral Transfection of Calcium Indicators in the Neonatal Mouse Brain

**DOI:** 10.3389/fncir.2018.00056

**Published:** 2018-07-23

**Authors:** Cynthia X. He, Erica D. Arroyo, Daniel A. Cantu, Anubhuti Goel, Carlos Portera-Cailliau

**Affiliations:** ^1^Department of Neurology, David Geffen School of Medicine at UCLA, University of California, Los Angeles, Los Angeles, CA, United States; ^2^Neuroscience Interdepartmental Program, David Geffen School of Medicine at UCLA, University of California, Los Angeles, Los Angeles, CA, United States; ^3^UCLA-Caltech Medical Scientist Training Program, David Geffen School of Medicine at UCLA, University of California, Los Angeles, Los Angeles, CA, United States; ^4^Department of Neurobiology, David Geffen School of Medicine at UCLA, University of California, Los Angeles, Los Angeles, CA, United States

**Keywords:** neuronal activity, cortex, development, GCaMP, *in vivo*, rAAV, sparsification, two-photon

## Abstract

The first three postnatal weeks in rodents are a time when sensory experience drives the maturation of brain circuits, an important process that is not yet well understood. Alterations in this critical period of experience-dependent circuit assembly and plasticity contribute to several neurodevelopmental disorders, such as autism, epilepsy, and schizophrenia. Therefore, techniques for recording network activity and tracing neuronal connectivity over this time period are necessary for delineating circuit refinement in typical development and how it deviates in disease. Calcium imaging with GCaMP6 and other genetically encoded indicators is rapidly becoming the preferred method for recording network activity at the single-synapse and single-cell level *in vivo*, especially in genetically identified neuronal populations. We describe a protocol for intracortical injection of recombinant adeno-associated viruses in P1 neonatal mice and demonstrate its use for longitudinal imaging of GCaMP6s in the same neurons over several weeks to characterize the developmental desynchronization of cortical network activity. Our approach is ideally suited for chronic *in vivo* two-photon calcium imaging of neuronal activity from synapses to entire networks during the early postnatal period.

## Introduction

The first three postnatal weeks in the mouse brain are of great interest to neuroscientists because they coincide with critical periods of experience-dependent plasticity (Petersen, 2007) and a phase of massive synaptogenesis (Micheva and Beaulieu, 1996; Holtmaat et al., 2009; Cruz-Martín et al., 2012). In particular, the time period around postnatal day (P) 12 is one of drastic sensory transitions as mice open their eyes, start whisking, and begin to actively explore their environment (Arakawa and Erzurumlu, 2015; van der Bourg et al., 2016). These developmental processes are critically important for understanding the causes of circuit dysfunction in a variety of developmental brain disorders, such as autism, schizophrenia, epilepsy, and intellectual disability.

In the last two decades, we have witnessed a large number of technological innovations for investigating neural circuits, from optogenetics and chemogenetics to rabies virus tracing and tissue clearing methods for fluorescence microscopy. Two of the most notable advancements have been the development of novel fluorescent calcium and voltage sensors, which allow researchers to record neuronal activity with synaptic resolution (Grewe and Helmchen, 2009), and the enhanced microscopy capabilities for imaging the anatomy and function of circuits across time in behaving animals (Wilt et al., 2009). These developments, combined with the ability to use a growing array of genetically encoded fluorescent molecules (e.g., fluorescent proteins, channels, pumps, etc.) via mouse genetics or viral transduction, now make it possible to trace the inputs and outputs of individual neurons and to record activity of specific neuronal cell populations in awake behaving mice.

The development of the ultrasensitive fluorescent genetically encoded calcium indicators (GECIs), especially GCaMP6 (Chen et al., 2013), has dramatically improved the action potential detection capability of two-photon calcium imaging. When combined with Cre-Lox genetics, this approach is particularly well suited for chronic recordings of neural activity in awake, behaving animals at the single-cell level, and in identified neuronal populations. The conventional approach for *in vivo* two-photon calcium imaging with GECIs typically consists of injecting a recombinant adeno-associated virus (rAAV) encoding the sensor at the time of implanting a cranial window over the desired cortical region. Imaging is typically performed 2–4 weeks after surgery to allow for sufficient expression of the virus (Tian et al., 2009; Chen et al., 2012, 2013; Zariwala et al., 2012). Unfortunately, this delay between injection and optimal GCaMP expression precludes calcium imaging experiments during the early postnatal period in rodents.

Overcoming this technological limitation would allow investigations to understand how experience shapes circuits during the first postnatal weeks and other important developmental milestones in the mouse brain. For example, previous calcium imaging studies demonstrated that sensory cortices undergo a rapid desynchronization of network activity at postnatal day (P) 12 (Golshani et al., 2009; Rochefort et al., 2009; Siegel et al., 2012). However, those studies employed synthetic calcium indicators that could only be imaged acutely for a few hours. Thus, the approach we outline here, which makes it possible to express GECIs (or other proteins) during early postnatal development and allows longitudinal imaging over several days to weeks in the same animals (for example, before *and* after P12), is a significant advance.

An alternative to the rAAV injection-based approach presented here is to use transgenic mice that express GCaMP. Unfortunately, in popular genetic mouse lines that drive expression of GCaMP, the promoters come online after this developmental period. For example, *Thy1*-GCaMP6 mice show stable expression of GCaMP6 in multiple cortical regions across months without apparent toxicity, but only after the third postnatal week (Dana et al., 2014). Similarly, the Ai38 mouse line expresses GCaMP3 when treated with tamoxifen at P7, but showed very low expression at 4 weeks of age (Zariwala et al., 2012). A different problem may occur when neuronal GCaMP6s expression is driven too early (during embryonic development), namely toxicity. For example, when we tried using *in utero* electroporation at embryonic day 16 with plasmids encoding GCaMP6s, we were able to use *in vivo* calcium imaging at around P7-9 to visualize a handful of neurons in layer (L) 2/3 that expressed GCaMP6s and showed synchronous activity. Just a few days later at P10-11, we could no longer find any GCaMP6s-expressing neurons, presumably due to neuronal cell death (unpublished observations). We surmise that the expression of GCaMP6s at early stages of neuronal differentiation or migration is irreversibly toxic to neurons, and therefore, a transgenic mouse line taking advantage of a promoter that would drive GECI expression during embryonic development is likely to be toxic to neurons.

Here, we describe a novel protocol for neonatal injection of rAAV encoding GCaMP6s at P1, which enables *in vivo* two-photon imaging of cortical neurons as early as P10. At P1, a modified burr hole surgery was used to inject the rAAV encoding GCaMP into the desired cortical area, and then at P8 or later, a cranial window was implanted over the previously injected area. Starting at day P11, repeated *in vivo* two-photon calcium imaging of layer (L) 2/3 neurons was possible with adequate GCaMP expression that persisted for weeks. We demonstrate the major advantage of this approach to chronically image the same population of neurons from P11 through young adulthood, allowing us to characterize the developmental desynchronization of cortical network activity and the evolution of sensory-evoked network during this critical period. We believe this approach will be valuable to the neuroscience community because the same neonatal viral injection approach could be used to express other genetically encoded calcium and voltage indicators, chemogenetic or optogenetic actuators, rabies virus tracers, or a variety of other fluorescent proteins, in different brain regions.

## Methods

Below, we provide detailed supply lists and step-by-step instructions for virus injections in newborn mice, as well as subsequent cranial windows. These protocols have been optimized for targeting of GCaMP6s to L2/3 neurons of barrel cortex for *in vivo* two-photon calcium imaging of spontaneous and whisker-evoked activity starting in the second postnatal week. Adjustments may be necessary for targeting deeper layers or different brain regions. For example, the choice of viral serotype and promoter will differ depending on the cell population being targeted. For our P1 injections, we used a rAAV1 with the synapsin promoter (AAV1.Syn.GCaMP6s.WPRE.SV40) (Chen et al., 2013), diluted to a working titer of 2E13 together with 1% filtered Fast Green FCF dye (to visualize the spread of the injection). Additionally, at E16 we used *in vivo* electroporation to introduce the pCAG-tdTomato plasmid to layer (L) 2/3 precursor cortical neurons.

### Animals

All experiments followed the U.S. National Institutes of Health guidelines for animal research, under an animal use protocol (ARC #2007-035) approved by the Chancellor’s Animal Research Committee and Office for Animal Research Oversight at the University of California, Los Angeles. We used male and female FVB.129P2 WT mice (JAX line 004828) and C57/BL6J mice (HSD C57Bl/6NHsd) housed in a vivarium with a 12-h light-dark cycle. Experiments were performed during the light cycle. Animals were weaned at P21-22 and afterward housed with up to five mice of the same sex per cage. Before P21, pups were housed with their dam.

### Reagents

All reagents were obtained from Sigma unless otherwise specified. All viral vectors were obtained from the University of Pennsylvania Vector Core.

- O_2_ tank.
- Artificial Tears eye lubricant ophthalmic ointment (Henry Schein, cat no. 048272).
- Sterile NaCl (Addipak, Teleflex cat no. 200-59).
- AAV vector encoding GCaMP (U. Penn virus core), diluted to 2e13 concentration with 1% Fast Green.
- 70% ethanol (Sigma-Aldrich, cat no. R3154-1GA).
- Betadine (Purdue Products, NDC 67618-155-16).
- Sterile Gelfoam (absorbable gelatin sponge) (Ethicon, Devine Medical cat no. MED-ETH1975).
- Cyanoacrylate glue (Krazy Glue, Office Depot, cat no. 366490).
- Ortho-Jet dental acrylic powder and liquid (Lang Dental, cat nos. B1330 and 1306).

### Drugs

- Lidocaine HCl 2% + epinephrine 1:200,000 (Fresenius-Kabl, cat no. 480927).
- Dexamethasone 2 mg/ml (Henry Schein, cat no. 002459).
- Carprofen (Rimadyl, 50 mg/ml, Zoetis).
- Isoflurane (Henry Schein, cat no. 029405). Note: Procedures using isoflurane should be conducted in well-ventilated areas, and should follow relevant animal care guidelines.

### Equipment

**FIGURE 1:**
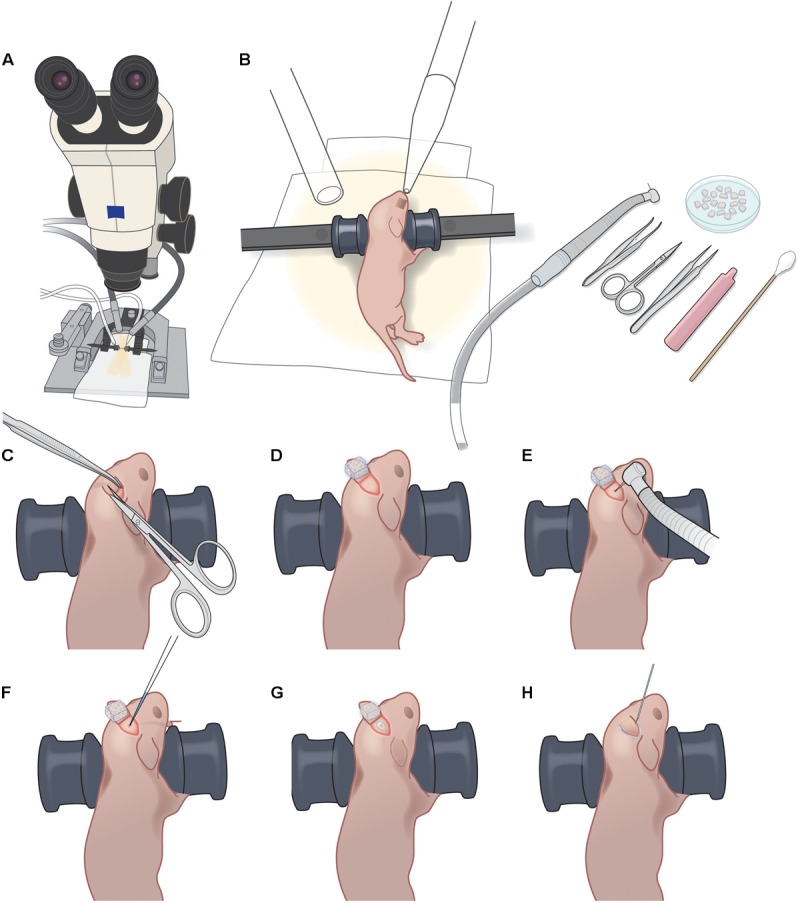
P1 injection setup and procedure. **(A)** Dissecting microscope with goose-neck illumination. **(B)** Positioning of P1 pup with blunt ear bars, isoflurane delivery and exhaust tubes, pneumatic dental drill, forceps, iridectomy scissors, fine forceps, sterile saline vial, sterile cotton swab, and petri dish with Gelfoam sponges soaking in sterile saline. **(C)**. Creating a 3–4 mm triangular skin flap over the desired injection area. **(D)** Folding back the skin flap and covering with wet Gelfoam to prevent the skin flap from drying out. **(E)** Light drilling of exposed skull to crack the bone slightly. **(F)** Injection of rAAV-GCaMP with glass micropipette. **(G)** Sealing of injection site with VetBond. **(H)** Sealing of skin flap with VetBond.

- Glass capillary puller (Narashige, model PC-10).
- Glass capillaries, O.D. 1.5 mm, I.D. 0.86 mm (Sutter Instruments, cat no. BF150-86-10).
- Picospritzer injection device (Parker Hannifin, model Picospritzer III).
- Glass bead sterilizer (Fine Science Tools, cat no. 18000-45).
- Water recirculating heating blanket (Stryker, cat no. 8002-062-012) and pump (Gaymar, cat no. 07999-000).
- Rodent trimmer (Wahl, cat no. 9962-717).
- Dissecting microscope (Zeiss, model Stemi 2000).
- Gooseneck light source (Dolan-Jenner MI-150, Edmund Optics cat no. 59-236).
- Stereotaxic frame and mouse adaptor (Kopf, cat nos. 900 and 926) (**Figure 1A**).
- Anesthetic vaporizer (Surgivet Classic T3) with airflow meter (Porter, cat no. GL-616).
- Induction chamber (VetEquip, cat no. 941443).
- Pneumatic dental drill (Midwest Tradition, Henry Schein, cat no. 7726063) with FG 1/4 carbide burr drill bits (Henry Schein, cat no. 100-7205).
- Dumont tweezers #4 and #5, straight dissecting scissors, 10 cm (World Precision Instruments cat nos. 500231, 14098, 14393).
- Small petri dish (35-mm diameter, Fisher, cat no. FB0875711YZ).
- Sterile cotton swabs (Henry Schein, cat no. 100-9175).
- Glass coverslips, 5 mm (Electron Microscopy Sciences, cat no. 72195-05).
- Needle tips, 18-gauge (BD, cat no. 305195).
- Titanium head bars (custom design: 0.125 × 0.375 × 0.05 inches).
- 2-photon microscope (custom built) (Cruz-Martín et al., 2010).
- Tunable Ti:Sapphire laser (Chameleon Ultra II, Coherent).
- Objectives, 4× (0.8 N/A) and 20× (0.95 N/A) water immersion (Olympus, UPLFLN 4×, XLUMPLFLN 20×).
- ScanImage (Pologruto et al., 2003).

### Set-Up

- Pipette preparation for injection: pull a custom micropipette from a 1.5 mm outer diameter/0.86 mm inner diameter glass capillary tube so that the length of the tapered tip is approximately 8 mm. Break pipette tip slightly by gently touching the tip to the side of sterilized forceps. The o.d. of the tip should be 12.5–25 μm. If the tip is too wide, the viral vector may reflux around the needle during the injection.
- Gelfoam preparation: use sterilized scissors to cut small pieces of Gelfoam, approximately 1 mm × 1 mm. Soak them in a small petri dish filled with sterile saline.
- Surgical instruments: sterilize instruments in a glass bead sterilizer and spray with ethanol before use.
- Heating blanket: turn on the water recirculating heating blanket 15 min before start of each surgical procedure.

### Step-By-Step Procedures

#### rAAV Injection at P1 (Timing 15–20 min)

1. *Preoperative care*: Administer Carprofen (5 mg/kg BW, s.c.) to the mouse. Note: we do not use dexamethasone for the virus injection in newborn pups.
2. Anesthetize the mouse with 5% isoflurane for induction, followed by 1.5–2% isoflurane for maintenance (vol/vol, via nosecone).
3. Use blunt ear bars to position the mouse on the stereotax so that the desired injection area is as flat as possible (**Figure 1B**). Ensure that the mouse pup remains warm (on the heating pad) throughout the procedure, and monitor breathing carefully, including tail and toe pinches. The ear bars should prevent movement of the pup during subsequent manipulations, without applying excessive pressure to its soft skull. Ensure that the heating blanket is functioning correctly. Note: newborn pups can stop breathing suddenly, making close monitoring under anesthesia crucial.
4. Sterilize operating field using three alternating wipes each of betadine and 70% alcohol.
5. Using the scissors, make one snip to create a 3–4 mm triangular skin flap over the desired injection area (**Figure 1C** and Supplementary Figure S1A). Fold back the skin flap and cover it with a piece of saline-soaked Gelfoam to prevent the skin from drying (**Figure 1D** and Supplementary Figures S1B,C).
6. Immediately apply a small drop of lidocaine/epinephrine onto the exposed skull. After 30 s, dry the surface of the skull with sterile cotton swab or dry Gelfoam.
7. Using the pneumatic dental drill, gently stroke the drill bit tip onto the skull surface to clear the periosteum. Use Gelfoam soaked in saline to clean the area of bone dust, then let the area dry. Note: do not push hard on the skull or the drillbit might pierce the bone and damage the dura/brain.
8. Once the exposed skull is clear of periosteum and dry, apply repeated light touches of the drill bit tip at the desired injection site until the bone has cracked enough to permit insertion of the glass micropipette (**Figure 1E** and Supplementary Figure S1D). Clean up any bleeding with moist Gelfoam. Ideally, there should be no bleeding. Note: if a large hole in the skull is created during the drilling, it will likely delay healing and result in a larger scar, which will make the later cranial window surgery less successful. The animal will likely not be suitable for imaging.
9. Load glass micropipette with approximately 200 nl of rAAV-GCaMP (working titer 2e13) with FastGreen and position for injection at a 45° angle to the skull surface.
10. Lower pipette until its tip has pierced through the cracked bone and into superficial cortex (**Figure 1F** and Supplementary Figure S1E). Inject rAAV using the Picospritzer using approximately 30 puffs of 3–5 ms durations at 40 PSI. Leave the pipette in place for 15 s and then withdraw pipette. Note: if fluid comes out of the skull surface around the pipette after withdrawal, it is an indication that its tip was not sufficiently small. The animal will likely not be suitable for imaging.
11. Using a needle tip (e.g., 18-gauge), apply a very small drop of VetBond to the injection site (just enough to seal the cracked area but not enough to reach the skin edges) and let dry completely (**Figure 1G** and Supplementary Figure S1F).
12. Replace the skin flap and seal the skin edges with a small amount of VetBond (**Figure 1G** and Supplementary Figures S1G,H).
13. Allow VetBond to dry before placing mouse in warm recovery cage. After the mouse completely recovers from anesthesia, return it to the litter. Carefully monitor the dam to ensure reintegration of the post-operative pup(s). Minimize rearrangements of the litter in the cage to minimize stress on the dam and reduce the possibility of cannibalism. Placing a small cardboard shed in the cage can also reduce stress for the dam.

#### Cranial Window Procedure at P8–P10 (Timing 45 min–1 h) (Modified Slightly From Cruz-Martín et al., 2010; Holtmaat et al., 2012)

1. Anesthetize the mouse with 5% isoflurane for induction, followed by 1.5–2% isoflurane for maintenance. Monitor anesthesia level throughout surgery by watching breathing, as well as using tail and toe pinches, and ensure that heating blanket is functioning correctly.
2. Use rodent trimmer to shave from the neck to the eyes, being careful not to trim any whiskers.
3. Use blunt ear bars to position mouse on stereotaxic frame, with anesthesia nose cone. The ear bars must be secured with just enough pressure such that the mouse’s head does not shift during surgery. The skull is still soft at P8-10 and care must be taken not to excessively squeeze the skull between the bars, as this will affect respiratory rate.
4. Administer Carprofen (5 mg/kg BW, s.c.) and dexamethasone (0.2 mg/kg BW, s.c.).
5. The original injection site should be apparent as a well-healed triangular scar on the skin, not raised or inflamed. If a large plug of granulation tissue is present at the original injection site and the skin is attached to the skull underneath, there is likely an excessive amount of scarring that will preclude a successful window surgery. The animal will likely not be suitable for imaging.
6. Sterilize the skin with three alternating wipes each of betadine and 70% alcohol.
7. Using the scissors, remove the skin on top of the skull, as well as the periosteum. Apply lidocaine/epinephrine to skin edges. After 30 s, dry the skull surface using cotton swabs.
8. Apply a small amount of cyanoacrylate glue to the skin edges, but do not apply glue over the bony sutures.
9. Use a pneumatic dental drill to very lightly carve a circular craniotomy, 3 mm in diameter. Apply lidocaine/epinephrine, let sit for 30 s, then dry skull. The skull at P8-10 is still very soft and the bone near the original burr hole may be particularly brittle. Drilling should proceed with minimal pressure to reduce the chance of sudden penetration and excessive bleeding.
10. Continue to gently drill along a circular groove until bone has been cracked all around the perimeter of the craniotomy. Clean up any bleeding on the bone with Gelfoam. The drilling can stop when the skull at the center of the craniotomy gives way as one pushes gently on it with the dental drill tip.
11. Soak the entire drilled area with saline using Gelfoam for at least 1 min. Then use fine-tipped forceps to gently lift the skull flap. Use Gelfoam to wipe away any residual scar tissue from the original burr hole and injection. Note: apply Gelfoam to stop any minor bleeding on the surface of the dura. Minimal bleeding at the edge of the window, if readily stopped with Gelfoam, will not impede imaging that same day. However, significant bleeding, bruising, or damage of the dura in the center of the window will preclude same-day imaging and, if severe, may also preclude subsequent imaging.
12. After the skull is removed use copious saline to irrigate the surface of the dura to remove bone dust. Note: if some bone dust remains, this will increase the chances of subsequent bone re-growth under the glass window.
13. While the dura is moist with saline, gently position a 3 mm glass coverslip over the craniotomy.
14. Hold the coverslip by applying gentle pressure on the center of the glass with the wooden end of a cotton swab (or with forceps) so that the glass is firmly resting against the bone edges. With the other hand, apply small drops of cyanoacrylate around the edges of the coverslip at two or three points, then drag it around the perimeter of the window. Cyanoacrylate should not seep under the glass and onto the dura. Let cyanoacrylate dry completely. Note: if some glue seeps under the glass, the animal will likely not be suitable for imaging.
15. Mix dental acrylic and apply over the entire skull surface around the window, sealing the edges of the coverslip. Use the same acrylic to secure a custom titanium headbar to an area of the head cap that will not interfere with the positioning of the microscope objective during *in vivo* imaging. The plane of the headbar should be parallel to that of the coverslip. Also use dental acrylic to make a small well around the window to hold water for the 20× immersion objective.
16. Allow the dental acrylic to cure for 5–10 min, and then move the mouse to a warm recovery cage. When the mouse has recovered from anesthesia completely, return to the litter. Monitor the dam closely to ensure the pups are safely reintegrated. Placing a cardboard shed in the cage can improve the dam’s caretaking of pups.
17. The mouse can be imaged later the same day, as long as the dental acrylic on the head cap and head bar is fully cured.

**FIGURE 2:**
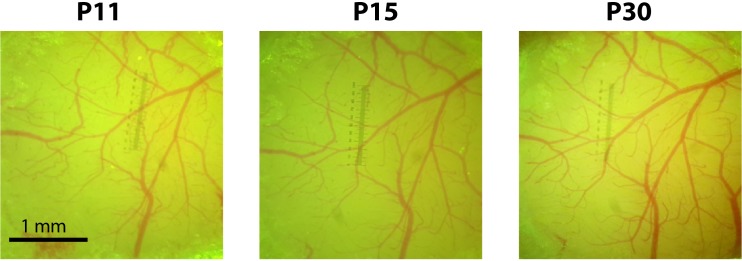
Cranial windows implanted at P10 remain optically transparent for weeks. Photographs of the cranial window at P11, P15, and P30 for a representative mouse injected with rAAV-GCaMP6s at P1.

Please also refer to Supplementary Table S1 for tips for improving injection efficacy while minimizing tissue damage and overall morbidity in newborn mice. Minimizing tissue damage and the resulting inflammation is key for successful cranial window implantation within 10 days of viral injection and to ensure subsequent window clarity (**Figure 2**).

### Electrophysiology

The brains were quickly removed and transferred to ice-cold artificial CSF (ACSF) containing (in mM): 119 NaCl, 2.5 KCl, 1.3 MgSO_4_, 1 NaH_2_PO_4_, 2.5 CaCl_2_, 26.2 NaHCO_3_, and 10 dextrose, bubbled with oxygenated 95% O_2_/5% CO_2_ to a final pH of 7.4. Acute coronal brain slices (300 mm) through the barrel cortex were cut on a vibratome (Leica VT1000S). The acute slices were incubated at 37°C in oxygenated ACSF for 1 h and then placed on a chamber maintained at 35–37°C on the stage of an upright Olympus BX51 microscope. Slices were submerged in ACSF, perfused at a rate of 2–4 ml/min, and bubbled with 95% O_2_/5% CO_2_. L2/3 pyramidal neurons were identified using differential interference contrast optics with a 60× 0.8 NA water immersion objective (Olympus). Cells were patched for 15–30 min and recordings were performed using whole-cell patch-clamp technique in current clamp configuration (Axon Instruments, Multiclamp 700B). Series resistances were manually compensated for standard patch pipettes (6–9 MΩ tip resistance) pulled on a Brown/Flaming microelectrode puller (Sutter Instruments, model P-97). Pipettes were filled with an intracellular solution containing (in mM): 130 K-gluconate, 5 KCl, 2 MgCl_2_, 10 HEPES, 4 Mg-ATP, 0.3 Na-GTP, 10 phosphocreatine, and 2 Alexa-488, adjusted to a final pH of 7.3. The ACSF solution in which slices were maintained was consistent between recordings with the exception of the [KCl], which ranged from 1.25 to 5 mM. The input resistance (*R*_m_) or membrane potential (*V*_m_) did not change more than 10% during the course of recording for each cell. All electrophysiological recordings were sampled at 10 kHz, digitized with custom written MATLAB software controlling an A–D board (National Instruments, PCI-6723), and saved for off-line analysis. All analyses were performed using custom-written software in MATLAB.

### *In Utero* Electroporation

*In utero* electroporation was performed as previously reported (Cruz-Martín et al., 2010, 2012). In short, pregnant female mice at gestation day E16 were anesthetized with isoflurane (5% induction, 1.5–2% maintenance vol/vol). A medial incision along the abdomen was made exposing the abdominal cavity. The uterine horns were gently exposed and each embryo was pressure injected with a plasmid encoding pCAG-tdTomato (500 ng/μl) in the left lateral ventricle with a Picospritzer. A set of three square pulses (50 ms duration, 35 V with 500 ms between each pulse) was administered to each embryo via a custom-built electroporator with the positive electrode paddle placed over the left somatosensory cortex. Throughout the procedure, the embryos were frequently irrigated with warm saline (37°C). The embryos were placed back inside the mother and the dam’s abdominal wall was sutured with absorbable sutures (muscle) and nylon sutures (skin).

### Histology

A 16-day-old mouse that underwent P1 injection was perfused intracardially with ice-cold 4% paraformaldehyde in 0.1 M phosphate buffer. The brain was then harvested and post-fixed overnight with 4% paraformaldehyde in 0.1 M phosphate buffer at 4°C. The brain was sliced in 100 μm-thick sections on a vibratome. Sections were mounted onto coverslips with Vectashield with DAPI (Vector Laboratories). Sections were imaged with an Olympus IX71, 10× obj with a N/A = 0.3.

### Optical Intrinsic Signal (OIS) Imaging

After the cranial window placement, OIS imaging was used at P16 to obtain whisker-responsive maps and confirm appropriate targeting of rAAV injection to the barrel cortex. Following a protocol previously described (Johnston et al., 2013), the contralateral whisker bundle was attached using bone wax to a glass needle coupled to a piezo-actuator (Physik Instrumente). Each whisker stimulation trial consisted of a 100 Hz sawtooth stimulation lasting 1.5 s. In order to delineate the cortical representation of whisker stimulation, the response signal was divided by the averaged baseline signal, summed for all trials, then thresholded at 50% of maximum response. OIS signal intensities were used solely for localization during calcium imaging and were not compared between animals.

### *In Vivo* Two-Photon Calcium Imaging in Head-Restrained Mice

Calcium imaging was performed on a custom-built two-photon microscope, with a Chameleon Ultra II Ti:Sapphire laser (Coherent), a 20× objective (0.95 NA, Olympus), and ScanImage software (Pologruto et al., 2003). Mice were lightly sedated with chlorprothixene (2 mg/kg, i.p.) and isoflurane (0–0.5%), and kept at 37°C with an electric heating blanket (Harvard Apparatus). Isoflurane was manually adjusted to maintain a breathing rate ranging from 100 to 150 breaths/min for P11-16 mice. Both spontaneous activity and whisker-evoked barrel cortex activity were recorded in the same mice at three postnatal ages, P11, P15, and P30. Whisker stimulation was delivered by bundling the contralateral whiskers (typically all macrovibrissae of at least ∼1 cm in length) to a glass needle coupled to a piezo-actuator with soft bone wax. Whiskers were stimulated for 1 s at 10 Hz with 10 s interstimulus intervals (i.s.i.), for a total of 10 stimuli (for network imaging) or with an i.s.i. of 5 s (for spine imaging). Whole-field images were acquired at 7.8 Hz (1024 × 128 pixels down-sampled to 256 × 128 pixels) for (network imaging) and 0.98 Hz for (spine imaging) (512 × 512 pixels). Spontaneous activity recordings lasted 60 s.

### Data Analysis for Calcium Imaging

Calcium-imaging data were analyzed using custom-written MATLAB routines, as described (He et al., 2017). All relevant data and MATLAB code are available upon request to the authors. X-Y drift in the movies was corrected using a cross-correlation-based, non-rigid alignment algorithm (Mineault et al., 2016). The choice of registration algorithm did not affect the data analysis, since the fluorescence data for each neuron was always normalized to its own baseline. A semi-automated algorithm (Chen et al., 2013) was used to select regions of interest, each representing a single-cell body, and extract the fluorescence signal (Δ*F*/*F*) for each neuron. A “modified *Z*-score” *Z*_*F* time series for each neuron was calculated as

$$Z\_F\left(t\right)=\frac{F\left(t\right)-mean\left(\begin{array}{cc} quietest & period \end{array}\right)}{std\left(\begin{array}{cc} quietest & period \end{array}\right)}$$

where the quietest period is the 10 s period with the lowest variation (SD) in Δ*F*/*F*. All subsequent analyses were performed using the *Z*_*F*(*t*) time series.

For analysis of aggregate activity within a particular time range, the mean of *Z*_*F*(*t*) within that time range was calculated for each ROI, and for each animal imaged, these means were compared across P11, P15, and P30. Only cells that had at least one calcium transient during the recording at all three postnatal ages were analyzed. To define whether an individual cell was “whisker-responsive,” i.e., showed time-locked responses to whisker stimulations, we used a probabilistic bootstrapping method, wherein we compared correlations between the stimulus time-course vs. the *Z*_*F* time series with correlations between the stimulus time-course and 1,000 scrambles of all calcium activity epochs in *Z*_*F*(*t*) (epoch = consecutive frames wherein *Z*_*F*(*t*) ≥ 3), as was previously described (He et al., 2017).

### Statistical Analyses

Graphs in **Figure 6** show data from neurons that were active across all time points P11, P15, and P30. For each ROI the *Z*_*F*(*t*) averages were calculated. To calculate statistical significance, Friedman’s two-way analysis of variance (ANOVA) by ranks for related samples was used with *post-hoc* pairwise comparisons corrected with Bonferroni adjustments. Pearson’s correlations were determined using custom MATLAB code and Friedman’s two-way ANOVAs by ranks were determined using SPSS 24 software (IBM). Graphs in **Figure 4** show all data points, as well as group medians. Based on the group sizes of *n* = 6, we used unpaired rank-based comparisons with bootstrapping (10,000 resamples), implemented using custom-written R code. Two-sided *p*-values were calculated, and the threshold for significance was set at *p* < 0.05.

## Results

**FIGURE 3:**
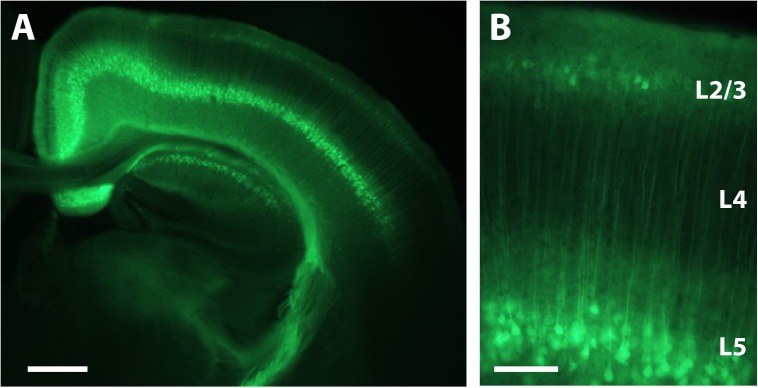
Expression of GCaMP6s in L2/3 and L5 after P1 injection. **(A)** Coronal view of a section through the brain of a P16 mouse that was injected with rAAV-Syn_GCaMP6s at P1. Note the spread of the expression across the hemisphere and the abundant expression in L2/3 and L5, but not L4. Scale bar = 500 μm. **(B)** Higher magnification image of GCaMP6s expression in L2/3 and L5. Scale bar = 100 μm.

We performed intracranial rAAV-Syn-GCaMP6s injections in anesthetized mouse pups at P1 through a small burr hole over primary somatosensory cortex, as described in the Materials and Methods (**Figure 1** and Supplementary Figure S1). When we used fluorescence microscopy of fixed tissue slices at P16 and later to survey the extent of GCaMP6s expression, we saw that there was no evidence of trauma from the injection, nor was there any obvious cortical pathology. In fixed brain tissue at P16 we could identify expression of GCaMP6s in both L2/3 and L5 neurons (but not in L4) throughout nearly the entire injected hemisphere (**Figure 3**).

**FIGURE 4:**
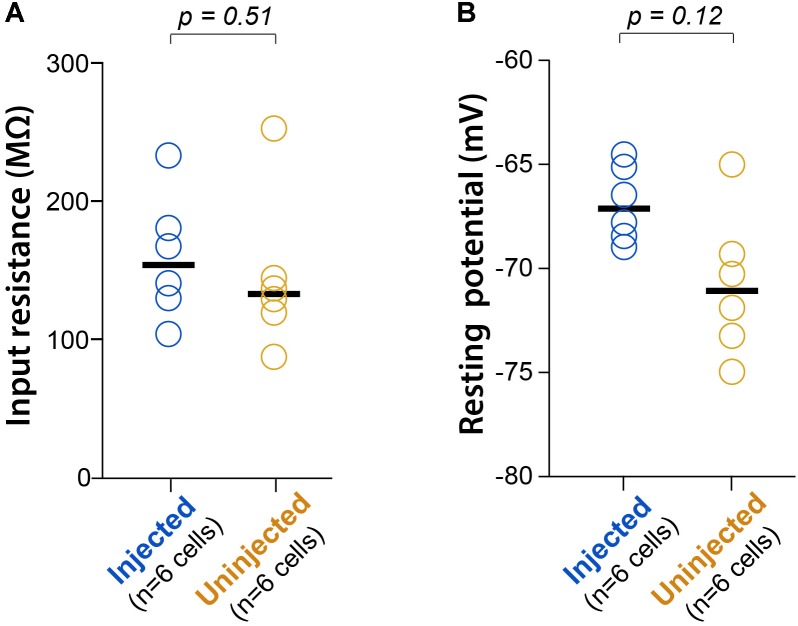
L2/3 neurons expressing GCaMP6s since P1 show normal electrophysiology. **(A)** Input resistance (*R*_m_) during whole-cell recordings of L2/3 neurons from P16-17 mice injected with AAV-GCaMP6s at P1, or from uninjected littermates (*n* = 2 mice each). Each circle represents data for one cell, bars represent group medians, and *p*-values are from unpaired rank-based two-group comparison with 10,000 resamples. **(B)** Resting membrane potential (*V*_m_) for the same comparison.

To confirm that neonatal GCaMP6 expression does not have cytotoxic effects on neurons, we used patch-clamp recordings in acute brain slices from two P16-17 pups that had been injected at P1, and two uninjected littermates at the same age, as previously described (Goel and Buonomano, 2016). We did not find statistically significant differences in either the input resistance (*R*_m_) or the resting membrane potential (*V*_m_) between GCaMP-expressing cells and cells from uninjected controls (*p* = 0.51 and *p* = 0.12, respectively; unpaired rank-based comparisons with bootstrapping; *n* = 6 cells per group) (**Figure 4**), suggesting that neonatal GCaMP expression does not have adverse effects on the neurons. We did not observe significant loss of neurons between P11 and P30 in the mice we imaged. However, with ongoing expression, we did observe a slight increase in the proportion of “filled-in” cells in areas of peak expression, closest to the original injection site. The percentage of all segmented soma ROIs that appeared “filled-in” was 2% at P11, 7% at P15 and 7% at P30. We did appreciate a slight decrease in the fraction of cells that demonstrated spontaneous activity, from 96% at P11 to 86% at P30.

To assess the suitability of neonatal viral injections for investigating cortical circuits in early postnatal mice, we used *in vivo* two-photon calcium imaging to record neuronal activity. We specifically chose to perform an experiment that could not have been possible without neonatal viral injections. We and others have previously demonstrated that cortical network activity undergoes a major network transformation at around the second postnatal week, such that neuronal activity becomes desynchronized (Golshani et al., 2009; Rochefort et al., 2009; Siegel et al., 2012; Gonçalves et al., 2013). However, those studies had recorded from different mice at different ages, so to this date, it had not been possible to record form an identified ensemble of neurons across time in a single animal. In order to achieve this, we injected three mice at P1 with rAAV-Syn-GCaMP6s.

Next, we permanently implanted glass-covered cranial windows at P8-10, following protocols we have previously established (Portera-Cailliau et al., 2005; Cruz-Martín et al., 2010, 2012). There are, however, important considerations when performing cranial window implants in mice previously injected with rAAV at P1. For example, the P1 injection can cause scarring in the injected area, which can make the subsequent cranial window surgery more difficult and reduce experimental efficiency. Scarring can be minimized by the following considerations during the virus injection: (1) a rapid surgical procedure with carefully calibrated anesthesia levels; (2) gentle burr hole drilling (with the drill at a 45° angle to the skull) that produces only a small crack in the skull through which the glass pipette can be smoothly inserted, without any visible signs of bleeding; (3) careful cleaning of the periosteum prior to and after the injection to prevent granulation tissue from forming at the site of injection, which would otherwise soften the bone and make the cranial window surgery more difficult; (4) careful application of the minimal amount of VetBond to seal the drilled area and, separately, the skin edges so that the skull and skin are not glued to each other. Injections done at P2 or later will drastically increase the chances of scarring. If there is scarring, then the subsequent cranial window surgery may be much less successful, reducing the experimental success rate. Following the recommendations above, as well as the steps in the Materials and Methods and the troubleshooting recommendations (Supplementary Table S1), can help ensure that windows remain clear for repeated imaging in previously injected neonatal mice (**Figure 2**).

**FIGURE 5:**
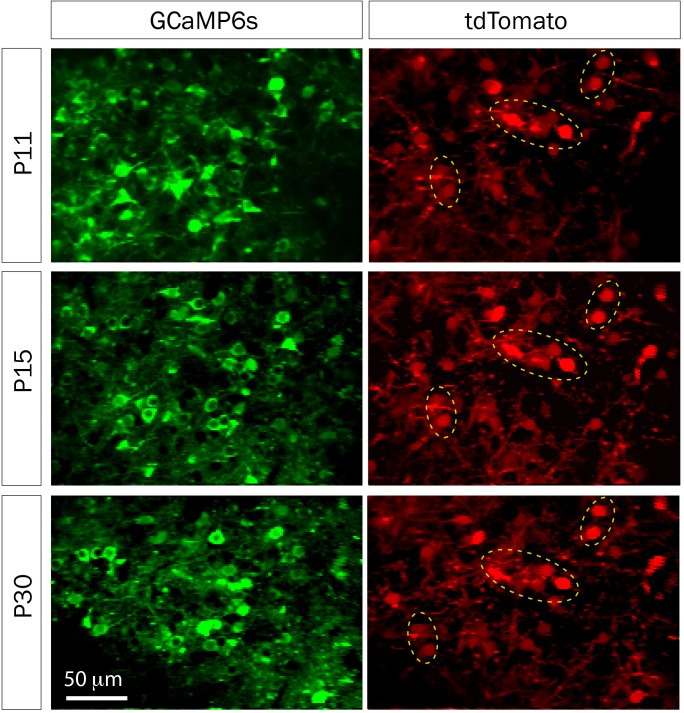
td-Tomato expression to help identify the same GCaMP6s field of view for calcium imaging. Representative fields of view of GCaMP6s and td-Tomato-expressing neurons in barrel cortex of a mouse at three different postnatal ages. This animal was electroporated *in utero* at E16 with a plasmid encoding the red fluorescent protein td-Tomato and then was injected with rAAV-GCaMP6s at P1. Expression of td-Tomato allowed us to identify the same field of view throughout postnatal development because of stable expression of clusters of neurons in the red channel (yellow dotted ellipses).

**FIGURE 6:**
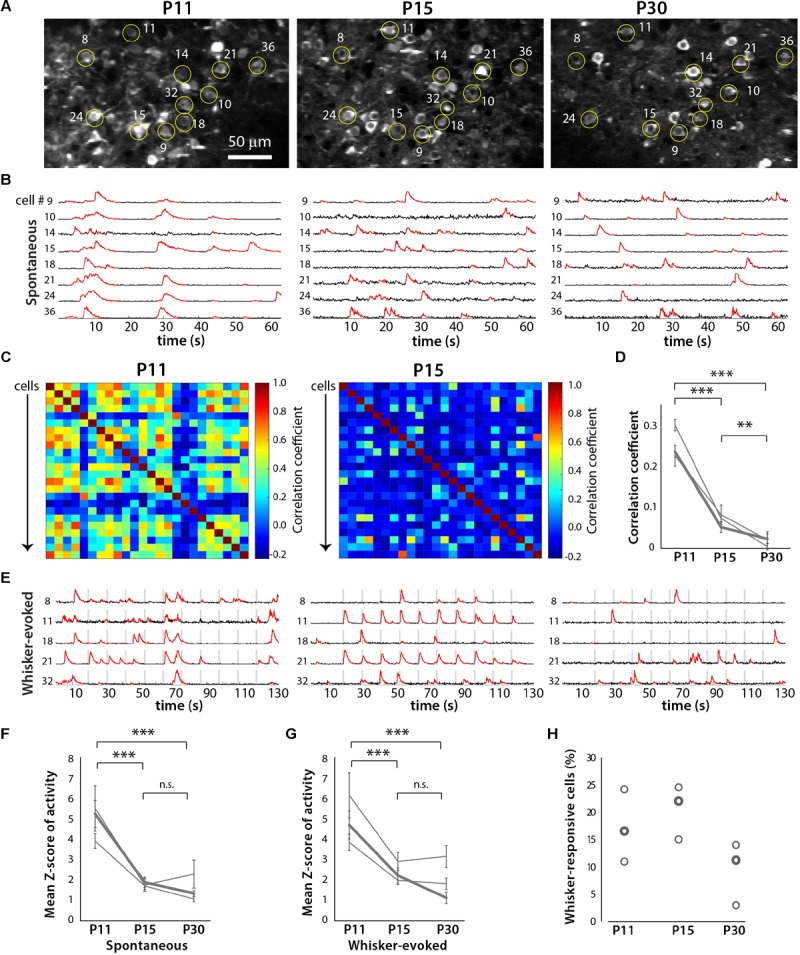
Developmental desynchronization of network activity in barrel cortex. **(A)** Example field of view for a two-photon imaging recording of GCaMP6s-expressing layer (L) 2/3 neurons in barrel cortex from a representative *in vivo* experiment at P11, P15, and P30 (*xyt* SD projection of 1,040 consecutive frames at 7.8 Hz). Example cells that could be identified at all three ages are labeled by yellow circles and numbers. For some of these cells, the corresponding fluorescence calcium traces are shown in panels **(B,E)**. **(B)** Spontaneous activity ΔF/F calcium traces of eight individual L2/3 neurons from the same field of view as in **(A)**, at P11, P15, and P30. **(C)** Correlation matrices displaying the correlation coefficients between the deconvolved calcium traces of all possible pairs of cells (*n* = 24) shown imaged in **(A)**. **(D)** Pair-wise correlation coefficients across developmental ages for three different mice. The bold line indicates data from the example recording shown in panels **(A–C,E)**. ^∗∗^*p* < 10^-3^, ^∗∗∗^*p* < 10^-5^. **(E)** Whisker-evoked activity ΔF/F calcium traces of eight individual L2/3 neurons from the same field of view as in panel **(A)**, at P11, P15, and P30. Vertical gray bars represent epochs of whisker deflection (10 Hz, 1 s duration, 10 s i.s.i.). **(F)** Magnitude of spontaneous activity (as determined by average *Z* scores of calcium traces). In panels **(F,G)**, the bold line indicates data from the example recording shown in panels **(A–C,E)**. ^∗∗∗^*p* < 10^-5^. **(G)** Magnitude of whisker-evoked activity (average *Z* scores). ^∗∗∗^*p* < 10^-5^. **(H)** Percentage of all L2/3 neurons imaged whose activity was time-locked to epochs of whisker stimulation (see Materials and Methods) for all three mice (*n* = 296, 401, and 248 neurons respectively).

In order to more easily identify the same ensemble of neurons over time, we used *in utero* electroporation to express td-Tomato in L2/3 neurons in S1 cortex (**Figures 5 and 6**). We performed *in vivo* calcium imaging of spontaneous network activity in slightly sedated, head-restrained mice from P11 to P30. At P11, L2/3 neurons in barrel cortex exhibit large but infrequent spontaneous calcium transients that are synchronous across the bulk of the neurons (**Figure 6B**). These giant network events have been previously described in acute cortical slices (Garaschuk et al., 2000; Allene et al., 2008) and *in vivo* (Golshani et al., 2009; Rochefort et al., 2009). In contrast, by P15, activity has become largely desynchronized, and this sparse firing of neurons prevailed at P30. To quantify the magnitude of this change in network behavior, we computed pairwise Pearson’s correlation coefficients for all possible pairs of neurons that had been imaged at all three ages from their deconvolved calcium traces (**Figures 6C,D**). The mean correlation coefficient of all cell pairs decreased significantly from 0.24 ± 0.01 at P11 to 0.06 ± 0.01 at P15 and 0.02 ± 0.01 at P30 (**Figure 6D**).

**FIGURE 7:**
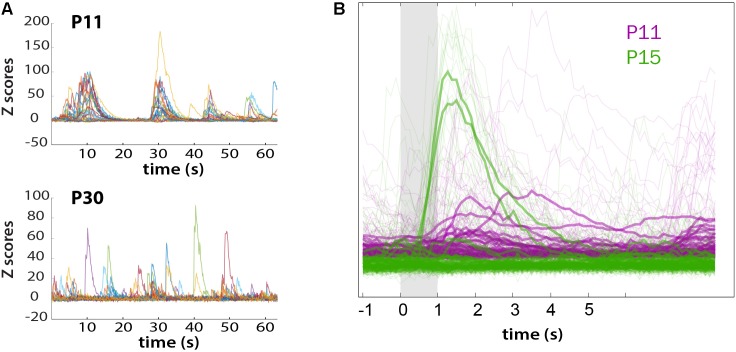
Loss of synchronous spontaneous activity of L2/3 neurons in developing barrel cortex coincides with their ability to respond to whisker stimuli between P11 and P15. **(A)** Overlay of fluorescent traces of spontaneous activity of the neurons in **Figure 6A** field of view. Different color traces are from different neurons (*n* = 24). **(B)** Neuronal activity is time-locked to whisker stimulation at P15, but not at P11. Traces were aligned to whisker stimulus and averaged over all 10 stimuli (bold traces).

We also recorded whisker-evoked activity at these same postnatal ages and observed clear whisker-evoked activity as early as P15 (**Figure 6E**). At P11, bursts of activity were so broad (sometimes lasting several seconds) that it was not possible to determine whether neurons were indeed responding to tactile stimulation. We also calculated the proportion of L2/3 neurons that responded to whisker stimulation in a time-locked fashion, as previously described (He et al., 2017), and found that the transition from a highly synchronous to desynchronized pattern of L2/3 activity between P11 and P15 coincides with the increase in responsiveness to whisker stimuli (**Figure 7**). We also found that the fraction of L2/3 neurons with activity that is time-locked to the epochs of whisker stimulation appears to decrease from P15 to P30 (**Figure 6H**).

**FIGURE 8:**
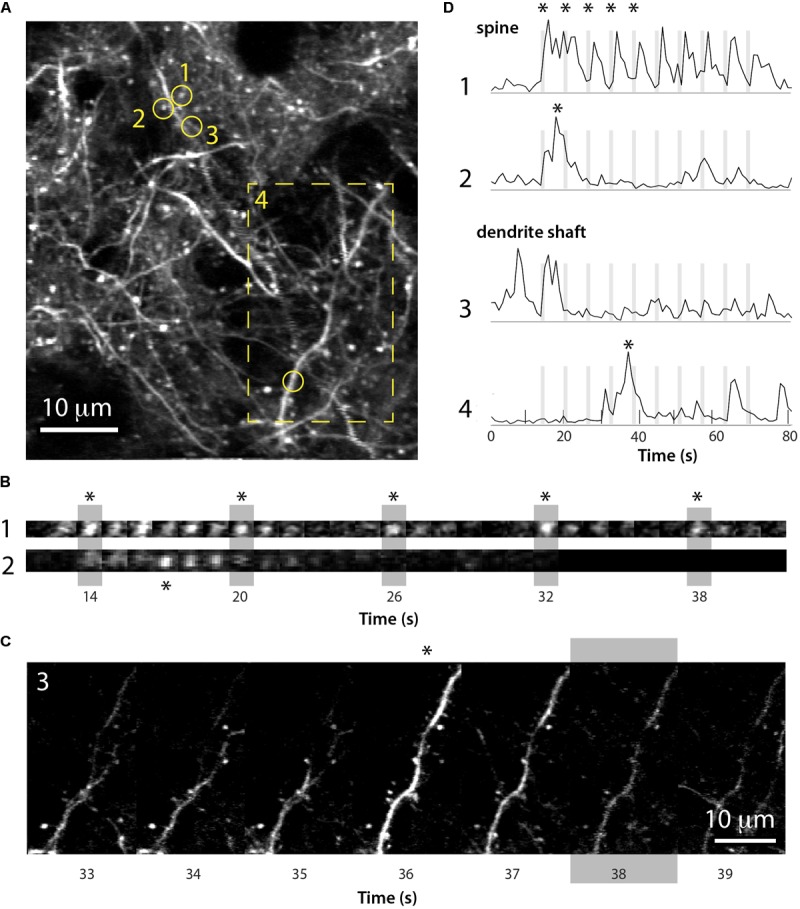
Imaging of GCaMP6s signals in dendritic spines of L2/3 neurons in barrel cortex at P15. **(A)** Field of view of apical dendrites from a representative experiment in a P15 mouse injected at P1 with rAAV-GCaMP6s (*xyt* st-dev projection of 80 slices over ∼80 s). Time-lapse images of calcium recording of spines 1 and 2 (circles) and dendrite 4 (boxed region) are shown in **(B,C)**. **(B)** Time lapse imaging sequence of dendritic spines 1 and 2 shown in **(A)** over five consecutive whisker stimulations (vertical gray bars). Each image is one frame (0.98 Hz image acquisition). Asterisks for each spine in **(B)** correspond to those in panel **(D)**. **(C)** Time lapse imaging sequence of the dendrite shown in **(A)** (box 4) over several seconds and a whisker stimulation (gray bar). Each image is one frame (0.98 Hz image acquisition). The asterisk above the time frame at 36 s corresponds to the asterisk in panel **(D)**. **(D)** Δ*F*/*F* traces for the calcium fluorescence signals for the dendritic spine and dendrite shaft regions of interest 1–4 shown in **(A)**, over 10 sequential whisker stimulations.

The neonatal viral injection approach also allowed us to record GCaMP6 signals in individual dendrites and dendritic spines of L2/3 neurons in early postnatal mice. We find that even by P15, individual dendritic spines display whisker-evoked activity (**Figure 8**). Interestingly, among neighboring spines within the same dendritic shaft some exhibited whisker signals, while others did not. Hence, this approach will be useful to unravel the role of individual dendritic spines in local circuit computations during early postnatal development.

## Discussion

We have presented a detailed description for a relatively straightforward protocol to express desired proteins in the neonatal mouse brain. Our postnatal injection protocol is very versatile, as it allows for successful targeting of specific cortical areas. For example, we recently used this neonatal virus injection approach to express GCaMP6s in barrel cortex of wild-type and *Fmr1* knockout mice (a model of Fragile X Syndrome), which allowed us to record the spontaneous and whisker-evoked activity of L2/3 neurons at P14-16 (He et al., 2017). This study revealed a novel cortical circuit defect in Fragile X mice, namely, the absence of adaptation in the activity of neurons to repeated whisker stimulation.

Our protocol does have some limitations. First, although we achieve GCaMP6s expression in L2/3 and L5, using the present rAAV1 and synaptophysin promoter, we failed to target expression to L4 neurons. Second, despite the fact that we could follow the same neurons from P11 to P30, it is admittedly difficult (if not impossible) to follow the entire population of neurons across these ages. For example, blood vessel growth within the developing cortex (which can obscure fluorescence from portions of the original field of view), or shifts in cell body position in the *z*-axis as the brain expands, prevented us from tracking the same cohort of cells. Third, there are considerable technical hurdles associated with performing successful, long-lasting cranial windows at P8-10, at the same site that was injected at P1. Scarring/healing around the injection site makes the skull more brittle, increasing the difficulty of the subsequent craniotomy. Additionally, whether the P1 injection was optimal or suboptimal (e.g., with excessive scarring) is not clear until the time of window placement. In our troubleshooting table (Supplementary Table S1), we further describe these technical issues.

There is some precedent for the use of neonatal injection of AAV vectors in neuroscience. A P0-1 injection of an AAV vector encoding Chronos-GFP into the rat visual cortex was used to enable optogenetic stimulation of visual cortex neurons with concurrent recording of thalamic activity (Murata and Colonnese, 2016). In mice, a P0 intraventricular injection of an AAV vector encoding YFP or Cre-tdTomato was shown to produce strong expression as early as P2, with levels of expression comparable to adulthood at P7 (Kim et al., 2013). However, the authors demonstrated the use of this approach only for structural imaging in fixed tissue, whereas we demonstrate benefits of longitudinal calcium imaging of network activity in living mice. Another paper used a similar approach to inject a rAAV1 encoding for GCaMP6f at P0-3 and performed *in vivo* two-photon calcium imaging of odor-evoked responses in olfactory bulb tuft cells (Cheetham et al., 2015). However, the earliest cranial window implantations and *in vivo* imaging in that study were done at P25, and only at a single imaging time point. The same could have probably been accomplished with rAAV injection at P10-15 at the time of the cranial window surgery. We demonstrate *in vivo* imaging of neocortical neurons starting 2 weeks earlier, at P11, and with repeated imaging of the same neuronal ensembles over a period of >2 weeks.

Previous studies have also used *in utero* electroporation in mice to transfect L2/3 and L5 precursors with channelrhodopsin and tDimer2, focusing on the prefrontal cortex and hippocampus (Bitzenhofer et al., 2017a,b), enabling manipulation of neuronal activity in the prefrontal cortex or hippocampus, as well as *in vivo* electrophysiology to characterize cortical oscillations in the P8-10 prefrontal cortex. We also routinely use *in utero* electroporation to express GFP or tdTomato in L2/3 precursors, but we did not find *in utero* electroporation of GCaMP6s to be successful beyond some scant expression at P7-9, suggesting to us that GCaMP6s may in fact be toxic to neuronal precursors. Whether prenatal or postnatal expression of a fluorescent indicator is ideal may, not surprisingly, depend on the specific indicator and the cell type being targeted.

Due to the technical challenges of performing such experiments in young mice, there is a relative dearth of studies that have recorded (or perturbed) brain network activity *in vivo* during the first two postnatal weeks, compared with similar studies in adults. Our P1 viral injection protocol is intended to help fill this gap, and therefore has a wide range of potential uses for neuroscientists, including those who are investigating the causes of circuit dysfunction in a variety of developmental brain disorders. Reasonable applications include the targeting of specific cell types via the use of Cre-Lox genetics (e.g., injecting AAVs encoding a Flexed constructs of GECIs or GEVIs into Cre driver mice); manipulations of circuit function at early postnatal ages via combination with optogenetic approaches, or with chemogenetics with designer drugs exclusively activated by designer receptors (DREADDs), as well as circuit mapping studies with rabies virus tracing; to be combined with *in vivo* recordings of neuronal activity in optically identified cells. Our approach could also be used to enable simultaneous *in vivo* imaging and behavioral assays in the same animals, in order to elucidate how the maturation of cortical circuit activity underlies the development of normal or aberrant (disease-related) behaviors.

## Data Availability Statement

All datasets generated and analyzed for this study are available upon request to the corresponding author.

## Author Contributions

CH and CP-C conceived the project and designed the experiments. CH and DC developed the P1 injection and imaging protocols and wrote the MATLAB code. CH, EA, and AG conducted the experiments and analyzed the data. CH, EA, and CP-C wrote the paper.

## Conflict of Interest Statement

The authors declare that the research was conducted in the absence of any commercial or financial relationships that could be construed as a potential conflict of interest.
