# Supplementary figures and images for: A Versatile Method for Viral Transfection of Calcium Indicators in the Neonatal Mouse Brain
Source: Front Neural Circuits. 2018 Jul 23;12:56. doi: 10.3389/fncir.2018.00056 (PMC6064716; doi:10.3389/fncir.2018.00056)

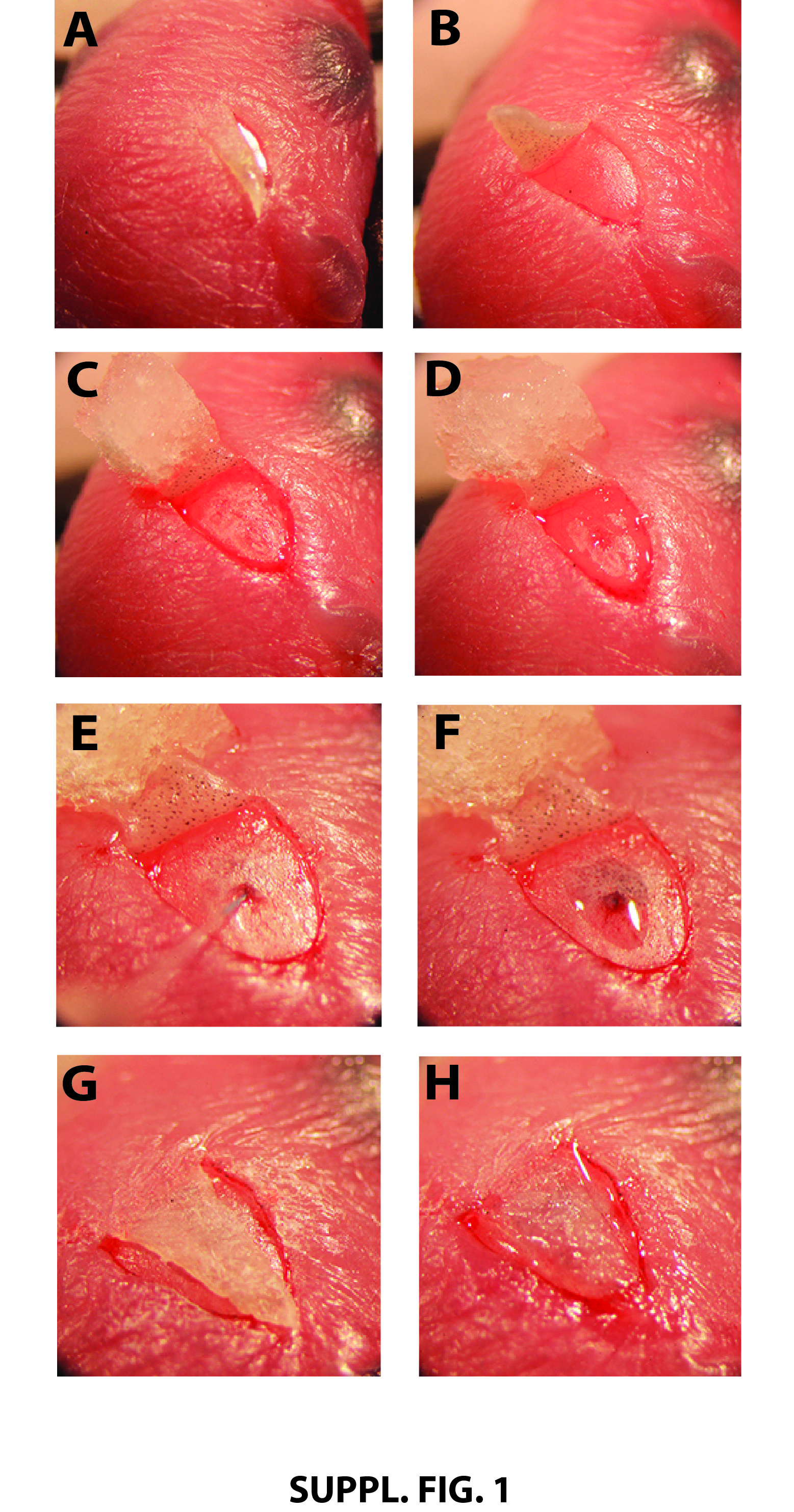

Supplement: Supplementary file 1 [file Image_1.JPEG]
