# Supplementary material for: A Versatile Method for Viral Transfection of Calcium Indicators in the Neonatal Mouse Brain
Source: Front Neural Circuits. 2018 Jul 23;12:56. doi: 10.3389/fncir.2018.00056 (PMC6064716; doi:10.3389/fncir.2018.00056)
Supplement: Supplementary file 2 [file Data_Sheet_1.pdf]

Supplementary Material

A versatile method for viral transfection of calcium indicators in the neonatal mouse brain

Cynthia X. He*, Erica D. Arroyo*, Daniel A. Cantu, Anubhuti Goel, and Carlos Portera-Cailliau

* These authors contributed equally to this work

Correspondence: Carlos Portera-Cailliau, M.D.-Ph.D: : cpcailliau@mednet.ucla.edu

Supplementary Table:

Suppl. Table 1: Troubleshooting for P1 injection and P10-13 cranial window

**Problem**	**Possible reason**	**Solution**
Mouse is moving	Insufficient anesthesia	1. Check that isoflurane delivery system: Check all tubing, valves, vaporizer settings.<br><br>2. Ensure nose cone immediately adjacent to or covering the nose.<br><br>3. Check that ear bars are not too tight and uncomfortable for the animal.<br><br>4. Increase maintenance anesthesia to 2.5%<br><br>5. If mouse is still moving, remove the mouse from the stereotax and re-induce at 5%, then reposition on stereotax.
Mouse gasping or dies during surgery	Excessive anesthesia, hypothermia, or excessive cranial pressure.	1. Adjust isoflurane level to ensure that mouse is still breathing regularly, though slowly, during surgery, and without response to toe or tail pinches.<br><br>2. Keep duration of surgery (anesthesia time)

		under 20 min.<br><br>3. Ensure that heating blanket temperature is appropriate.<br><br>4. Check that ear bars are not too tight. Skull should be immobile but only barely bulging between the ear bars.
Bleeding during drilling	Damaged blood vessels, or the drill bit punctured the dura.	1. Apply wet Gelfoam to the drilled area and let sit for 30-60 s. If bleeding stops, then proceed with surgery. If bleeding does not stop, irrigate with sterile saline and then apply wet Gelfoam.<br><br>2. If the drill bit has clearly punctured through the skull and into the dura, terminate the experiment.
Vector does not exit the pipette tip	The pipette is clogged	1. Increase the Picospritzer pulse duration and apply a single larger pulse of pressure to try and unclog the pipette; then proceed with the injection at lower duration.<br><br>2. If the pipette tip remains clogged, retract the pipette and switch to a new pipette.
rAAV solution refluxes around pipette tip emerges from burr hole	The pipette tip is too wide	1. Avoid using pipettes with a large tip diameter; Use minimal pressure to break the pipette tip or by gently touching to moistened Kimwipe.<br><br>2. Reduce the Picospritzer pulse duration and increase the pause time between pulses (i.e., reduce the volume injected per pulse), to allow time for the injected solution to be absorb around the pipette tip.<br><br>3. After injection is complete and the pipette is retracted, use wet Gelfoam to thoroughly clean the skull surface before applying VetBond.
Skin at injection site has adhered to the skull (excessive scarring)	Removal of too much bone, excessive VetBond application, or skin flap replacement	1. If the skin at the injection site is adhered to the skull, a good cranial window surgery can still be performed (i.e., the dura and skull may not be adhered).<br><br>2. If the skull flap cannot be lifted due to

	before VetBond on skull was dry	adhesion, terminate the surgery.
Bleeding occurs upon bone flap removal	Blood vessel damage or dura damage	1. Apply Gelfoam soaked in saline to the drilled area and let it sit for 30 s. If bleeding stops, proceed with surgery. If bleeding does not stop, irrigate with sterile saline and then apply wet Gelfoam.<br><br>2. If the drill bit has clearly punctured through the dura, or if the bone flap has ripped the dura, terminate the surgery.

Supplementary Figure:

Suppl. Figure 1: Surgical photographs of P1 injection procedure.

A. Creating a 3-4 mm triangular skin flap over the desired injection area.
B. Folding back the skin flap.
C. Covering the skin flap with wet Gelfoam to prevent drying and shrinking; exposed skull is dry after light scraping with dental drill.
D. Small crack in skull after drilling.
E. Injection of rAAV-GCaMP with glass micropipette.
F. Sealing of injection site with VetBond.
G. Replacement of skin flap.
H. Sealing of skin flap with VetBond.

frontiers
